# Analysis of the stability of the reference genes GAPDH, SDHA and RPL-19 in sheep from a semi-arid region infected by gastrointestinal nematodes

**DOI:** 10.1186/s12917-023-03709-x

**Published:** 2023-09-07

**Authors:** Jorge Lucas Nascimento Souza, Fernanda Cavalcante Silva, Carlikelly Gleicy da Silva, Isabela Maria Fortaleza Neves Bomfim, Henrique Rocha de Medeiros, Lilian Giotto Zaros

**Affiliations:** 1 Graduate Program in Parasitary Biology, Department of Microbiology and Parasitology, Federal University of Rio Grande do Norte, Natal, Rio Grande do Norte Brazil; 2 Graduate Program in Animal Production, Jundiaí Agricultural School, Federal University of Rio Grande do Norte, Macaíba, Rio Grande do Norte Brazil; 3 Graduate Programan in Biological Sciences, Biosciences Centre, Federal University of Rio Grande do Norte, Natal, Rio Grande do Norte Brazil; 4 Jundiaí Agricultural School, Federal University of Rio Grande do Norte, Macaíba, Rio Grande do Norte Brazil; 5 Department of Microbiology and Parasitology, Biosciences Centre, Federal University of Rio Grande do Norte, Natal, Rio Grande do Norte Brazil

**Keywords:** GAPDH, Gastrointestinal tract, Sheep and RT-qPCR

## Abstract

Analyzing the stability of reference genes already described as universal is an important methodology to lead gene expression analysis because different studies have shown that the expression of universal reference genes may vary between experimental treatments. In this sense, the glyceraldehyde 3-phosphate dehydrogenase (GAPDH), Succinate dehydrogenase complex subunit A (SDHA) and Ribosomal Protein L-19 (RPL-19) reference genes (already described in other studies with sheep from different regions, breeds and infectious agents or in organisms evolutionarily close to sheep) were investigated in the abomasum, small and large intestines of resistant and susceptible crossbred sheep groups to gastrointestinal nematode infections in the Semi-arid region in Northeast of Brazil. The animals were naturally infected to determine the resistance or susceptibility status by counting eggs per gram (EPG) of feces from the gastrointestinal tract after 33 weeks of observations of infection evolution. Relative gene expression was performed by RT-qPCR methodology using Sybr green and relative gene expression stability was tested by different software programs such as REST, BestKeeper, geNorm and Normfinder. Our results showed the susceptible animals had increase in egg counts per gram of feces than resistant animals (p < 0.001), and both groups showed a mixed infection by nematodes of the genus *Haemonchus*, *Trichostrongylus*, *Oesophagostomum* and *Trichuris*. Furthermore, we show the importance of analyzing different genes in different software programs and the importance to choose ideal reference genes. In this sense, GAPDH was the most stable gene in the abomasum, whereas SDHA was the most stable in the small and large intestines. In addition, we discuss about variables which can interfere in relative expression such as breed, species, climate and tissue. However, utilizing other reference genes already described in other studies with the same and different variables should be performed.

## Background

Sheep farming is a greatly important socioeconomic activity, especially in the semi-arid regions of Brazil in the Northeast region of the country [1, 2]. Small ruminant animals, especially sheep, are severely affected by numerous factors such as gastrointestinal nematode infections. This is one of the major causes of losses in sheep production and negatively impacts the health of infected sheep due to weight loss of the animals and a drop in productivity, which in turn can evolve to death and cause considerable economic impact in the agribusiness sector [3–6]. The main control strategy for gastrointestinal nematodes is the use of anthelmintic drugs, however this has become less effective due to the development of anthelmintic resistance, specifically in Brazil [7–11].

Therefore, new control strategies should be adopted such as selecting more resistant hosts to parasites [12, 13]. Animals have genetic differences and therefore respond differently to these infections, they may be resistant, resilient or susceptible to gastrointestinal nematode infections [14, 15] by activating different lymphocyte T Helper (Th) subsets proliferations which secrete different cytokines [13, 16, 17]. Thus, it is necessary to identify the genes associated in its process and know the gene expression in order to understand the mechanisms involved in immune response to these parasites [18].

RT-qPCR has been used in ruminant cytokine gene expression studies [13, 17–21] Nevertheless, variations in these assays caused by the amount of starting material, RNA integrity, enzymatic efficiency and differences between tissues and cells in overall transcription activities are some variables which can interfere in gene expression analysis studies and must be controlled. Therefore, strategies such as reference genes [18, 22] are suitable to decrease these variations when an ideal reference gene is chosen and used in these assays [23].

Most studies show validation of reference genes around the world [24–29]. However, most studies in Brazil have reported the use of reference genes in bovine models [12, 16, 30], and there are only two studies [17, 18] which show its importance in sheep models. In this sense, it is essential to analyze whether these previously reported reference genes used in sheep and other genetically similar ruminants models with different or the same variables such as different regions, infectious agent and tissues can be used in sheep from the semi-arid region in Northeast Brazil. Thus, the aim of this work was to evaluate the relative gene expression stability of three reference genes, namely glyceraldehyde phosphate dehydrogenase (GAPDH), succinate dehydrogenase complex subunit A (SDHA) and Ribosomal Protein L 19 (RPL-19), already described using other breeds, different climates and other ruminants similar to sheep, but in Brazilian crossbred sheep which are resistant and susceptible to gastrointestinal nematode infections.

## Materials and methods

### Animals, sample collection and processing

All experiments were performed in accordance with the norms issued by the National Council for the Control of Animal Experimentation (CONCEA) and approved by the Committee on Ethics in the Use of Animals of the Federal University of Rio Grande do Norte (CEUA/UFRN), under protocol 022/2015. The sheep used in this study were born and raised in the experimental area of Unidade Acadêmica Especializada em Ciências Agrárias (UAECIA) belonging to the Universidade Federal do Rio Grande do Norte, in Macaíba, Rio Grande do Norte state, Northeast Brazil (5° 53’ 34’’ S and 35° 21’ 50’’ W). Among the born sheep, we selected a herd of 36 sheep (crossbreed ½ Santa Inês x ½ crossbreed sheep) at four months old and were kept without anthelmintic treatment for 33 weeks on the same *Panicum maximum* cv. Massai pasture naturally contaminated by eggs and infective larvae of gastrointestinal nematodes in the UAECIA. The animals were monitored weekly to collect feces samples. These were collected directly from the animals’ rectal ampulla, processed with a hypersaturated solution and eggs are counted in a McMaster chamber according to the descriptions of Ueno and Gonçalves [31]. After this period, six resistant animals (i.e, group with lowest FEC average) and six susceptible animals (i.e, group with highest FEC average) and with the lowest amplitudes during the experimental period were classified as the resistant and susceptible group, respectively, and were slaughtered using a nonpenetrating captive dart followed by sectioning of the jugular veins and arteries for bleeding and abomasum, small and large intestine samples were collected and immediately inserted in RNA later solution, transported to the laboratory and then stored and maintained at -80 °C for total RNA extraction.

### Total RNA extraction and cDNA synthesis

Total RNA was extracted according to Chomczynski and Sacchi [32] using Trizol Reagent (Invitrogen Co., Carlsbad, CA, USA) individually for each animal organ. Briefly, 1mL of trizol was added to 1 gram of tissue, broken into small pieces and vortexed homogenized. Then, 200µL of chloroform was added, followed by vigorous stirring, incubated for 5 min at room temperature 25 °C and centrifuged (16.000 XG, 15 min, 4 °C) and the aqueous phase was transferred to other tubes, and 500µL of isopropanol were added and carefully homogenized, followed by incubation for 10 min at room temperature. Then, the samples were centrifuged (13.000 XG, 10 min, 4 °C). The pellet was washed with 1mL of 75% ethanol and centrifuged (10.500 XG, 5 min, 4 °C). The RNA precipitate was dried at room temperature and eluted at 30µL in DEPC water.

The RNA concentration was assessed by spectrophotometry (NanoDrop one, Thermo Fisher Scientific) at OD 260 nm and the purity was checked by the OD 260/280 ratio. The RNA integrity was verified by desnaturing agarose-gel electrophoresis. The complementary DNA (cDNA) was reverse transcribed from 5 µg of total RNA by using SuperScript™ IV First-Strand cDNA Synthesis Reaction Kit (Invitrogen/Life Technologies). Briefly, 5 µg of RNA was added to the kit’s reaction mix (primer oligo dT and dNTPs) and incubated at 65 °C for 5 min. Subsequently, another mix was added (SSIV Buffer, DTT, Ribonuclease Inhibitor and Enzyme from the kit) incubated at 55 °C for 10 min and then incubated at 80 °C for 10 min. Then removal of other strands of RNA in each sample was performed, from the addition of 1µL *E. coli* RNase H and incubated at 37° for 20 min.

### Reference genes selection

GAPDH (glyceraldehyde 3-phosphate dehydrogenase), RPL-19 (ribosomal protein L 19) and SDHA (succinate dehydrogenase complex subunit A) reference genes were tested. These genes were selected based on commonly used for RT-qPCR studies in different ruminant tissues. Primer sequences were obtained in studies involving sheep or bovine from Zaros et al. [16] for GAPDH and RPL-19 and SDHA from Smeed et al. Primer sequences and amplicon lengths are shown in Table 1.

**Table 1:** Primer sequence, amplicon sizes, PCR conditions, Melting temperature, slope values, correlation coefficients (R^2^) and Efficiency values (E) for each primer pair

Gene	Sequence	Amplicon (pb)	Annealing (°C/s)	Extension (°C/s)	Melting (°C)	Slope	R^2^	E
**GAPDH**	F: GGCGTGAACCACGAGAAGTATAA R: CCCTCCACGATGCCAAAGT	119	57 °C/30s	72°/8s	83,5	-3,345	0.998	1.99
**SDHA**	F: ACCTGATGCTTTGTGCTCTGC R: CCTGGATGGGCTTGGAGTAA	126	57 °C/30s	72°/8s	85,3	-3,580	0.990	1.90
**RPL-19**	F: GAAATCGCCAATGCCAAC R: GAGCCTTGTCTGCCTTCA	361	51 °C/30s	72°/22s	85,5	-3,770	0.970	1.84

### Quantitative PCR (qPCR), primer specificity and amplification efficiency

The qPCR was performed for each gene using a 7500 fast Real-Time PCR system (Applied Biosystems™) with the same initial denaturation and denaturation step for all primers (94 °C for 2 min and 94 °C for 15 s, respectively) according to the manufacturer’s instructions with the PowerUP™ Sybr green master mix kit (Applied Biosystems™). Duplicates of each sample were included for each experiment in addition to a non-template reaction (negative control). The fluorescence acquisition temperature was 72 °C for all genes, Threshold cycle (Ct) values were determined at the same fluorescence threshold line for all the genes, and the Ct values for each sample was obtained by calculating the arithmetic mean of duplicate values. Each sample volume was analyzed consisting of 5 µL from the kit, 2.0 ng of cDNA, 10pmol forward and reverse primers, and Milli-Q Water to complete the final volume of 10 µL.

After confirming the ideal conditions previously tested for each gene, the specificity of the amplified products was evaluated by melting curve analysis and cDNA serial dilutions of amplified products, in addition to confirmation by visualizing the expected amplicon size in 2% agarose gel electrophoresis. A melting curve analysis was performed for all genes studied in the same conditions (60 to 95 °C at 0.1 °C/s). Melting temperature (Tm) was identified by the highest peak of the curve (midpoint) and serial cDNA dilution curves were produced to calculate the amplification efficiency for all genes. Finally, a linear regression was performed to determine the slope of the stretch to determine the amplification efficiency (E), which is necessary to use in formula: E = 10 ^(−1/ slope)^ [33].

### Stability test, software description and statistical analysis

The stability of reference gene expression was tested using the methodologies described in four computational software programs: REST© (Relative Expression Software Tool) which tests the significance of the transcripts between groups by non-parametric analysis using the Pair Wise Fixed Reallocation Randomization Test (Ratio for expression between groups must be close to 1) [33]; Bestkeeper which calculates the Standard Deviation [± Crossing Point] for each gene from the Ct value and efficiency amplification value (Lower Std. Dev. [± CP] values indicate greater stability) [34]; geNorm which calculates the stability expression means (M value) of each gene (Lower M values indicate greater stability) [23]; and Normfinder, a software which provides stability values for each gene (lower stability values indicate the highest stability) [22]. The geNorm and Normfinder programs suggest associations between genes to use and consider values from relative quantification (E^−ΔCt^) to analyze, being different from REST and Bestkeeper which use Ct values.

To detect possible outliers, Grubbs’ test was used. The Kolmogorov-Smirnov test was used to verify data distribution. The Ct values were expressed as arithmetic means (Standard deviation and coefficient of variation) between tissues. The *t* test with Welch’s correction was used to perform comparisons of the gene expression in each reference gene between groups in each tissue and comparision of EPG between resistant and susceptible groups. The GraphPad Prism 8.0.1 software (GraphPad Inc, USA) was used in these analyzes and to plot values obtained from other programs. In addition, the significance level was set at p value equal to or less than 0.05.

## Results

### Parasitology

**Fig. 1:**
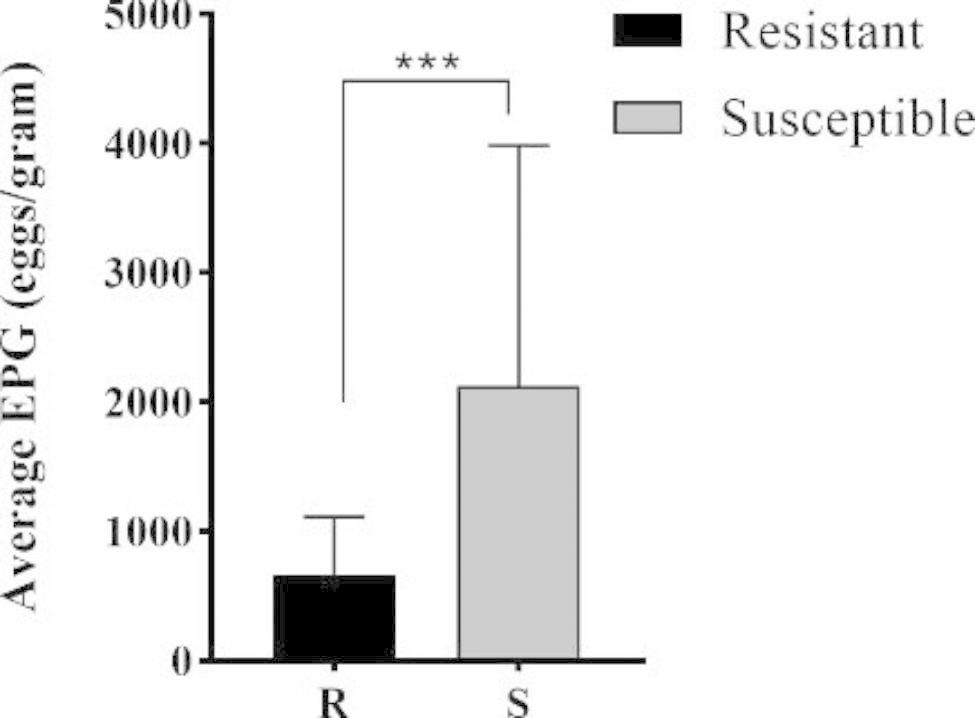
Average Egg Per Gram (EPG) of feces of the resistant (R) (n = 6) and susceptible (S) (n = 6) groups obtained from the 33 weeks experimental period. *t* test with Welch’s correction was used to compare groups. The results are shown as the mean ± SD and were represented by ***p < 0.001

After the 33 weeks the animals were classified in the resistant group showed mean EPG values ​​of 669.80 (± 444.1) and susceptible group showed mean EPG values ​​of 2123.44 (± 1864) (p < 0.001) (Fig. 1). All recovered eggs were identified as eggs of the Trichostrongylidae family. For this reason, we performed the total recovery of 926 adult parasites at necropsy. *Haemonchus contortus* was the most prevalent parasite (73,22%), followed by *Trichostrongylus columbriformis* (20,30%) and finally *Oesophagostomum columbianum* (4,32) and *Trichuris ovis* (2,16%). Thus, the EPG and identification of adult parasites allowed the confirmation of the natural infection of the animals.

### Specificity, efficiency, conditions and reference gene expression profiles in different tissues

All data demonstrated specificity in the PCR reactions. The relationship between threshold cycle (Ct) and the log copy number of serial dilution cDNA for all genes was linear with an R^2^ ranging from 0.97 to 0.99, indicating that Ct values changed proportionally to the serial dilution of the samples. Efficiency values ranged from 1.84 to 1.99, indicating efficient amplification near the theoretical optimum level of 2 [35]. Melting curves presented a single peak indicating the absence of primer-dimer formation during the reaction and amplification specificity. Informations described for each primer pair are shown in Table 1.

**Fig. 2:**
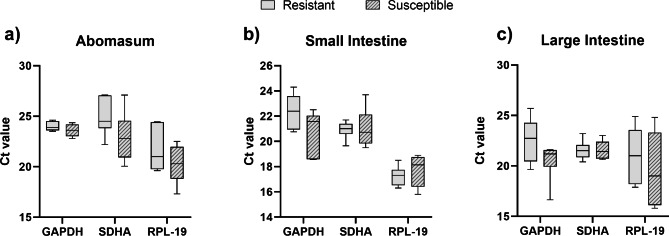
Distribution of expression levels based on Ct values from each reference gene separated by group (resistant and susceptible) in the three evaluated tissues. No samples indicated a significant difference (p ≤ 0.05) by the *t* test with Welch’s correction

Ct values for the three tissues ranged from 21.54 to 23.80 for GAPDH, 20.98 to 23.95 for SDHA, and 17.48 to 20.98 for RPL-19. The lower Ct value for RPL-19 indicated that this gene reached the detection threshold with less amplification cycles than GAPDH and SDHA, and thus it was more abundant in the small intestine, large intestine and abomasum, respectively (Table 2). However, the coefficient of variation in the three tissues ranged from 5.87 to 16.64 for RPL-19, 2.29 to 10.43 for GAPDH, and 4.12 to 9.67 for SDHA across all the tissues and groups. Thus, GAPDH in abomasum and SDHA in small and large intestine presented the lowest coefficient of variation (Table 2). Reference gene expression level comparisons between the resistant and susceptible group to gastrointestinal nematode infections in different tissues presented no significant difference (p > 0.05) (Fig. 2).

**Table 2:** Analysis of GAPDH, SDHA and RPL-19 reference genes in the abomasum (AB), small intestine (SI) and large intestine (LI) from resistant and susceptible groups. Data shown represent the Ct mean ± SD.

Gene	AB		SI		LI		
	Ct mean ± SD	CV (%)	Ct mean ± SD	CV (%)	Ct mean ± SD		CV (%)
**GAPDH**	23.80 ± 0.55	2.29	21.58 ± 1.74	8.06	21.54 ± 2.25		10.43
**SDHA**	23.95 ± 2.32	9.67	20.98 ± 1.11	5.29	21.56 ± 0.89		4.12
**RPL-19**	20.98 ± 2.08	9.93	17.48 ± 1.03	5.87	20.32 ± 3.38		16.64

### Stability test and identification of the best reference gene in each tissue

According to REST software, GAPDH showed a lower expression variation (1.3x) between the resistant and susceptible groups in the abomasum than SDHA (4.0x) and RPL-19 (2.7x); SDHA in the small intestine showed a lower expression variation (0.93x) between the resistant and susceptible groups than RPL-19 (0.7x) and GAPDH (2.9x); SDHA also showed a lower expression variation in the large intestine (0.98x) between the resistant and susceptible groups than RPL-19 (2.7x) and GAPDH (4.0x). Therefore, GAPDH was ranked as the most stable gene in abomasum, followed by SDHA in the small and large intestine, and lastly RPL-19 in both the resistant and susceptible animals. A significant difference (p < 0.05) was observed for SDHA in the abomasum and GAPDH in the small and large intestines (Table 3).

**Table 3:** Analysis of GAPDH, SDHA and RPL-19 reference genes in the abomasum (AB), small intestine (SI) and large intestine (LI) from resistant (R) and susceptible (S) groups by REST software. Asterisks indicate a significant difference between the resistant and susceptible groups (p ≤ 0.05)

Gene	AB		SI		LI		
	Expression (R/S)	p	Expression (R/S)	p	Expression (R/S)		p
**GAPDH**	1.33	0.087	3.00	0.022*	4.070		0.025*
**SDHA**	4.02	0.030*	0.930	0.811	0.983		0.953
**RPL-19**	2.75	0.081	0.724	0.251	2.701		0.321

**Fig. 3:**
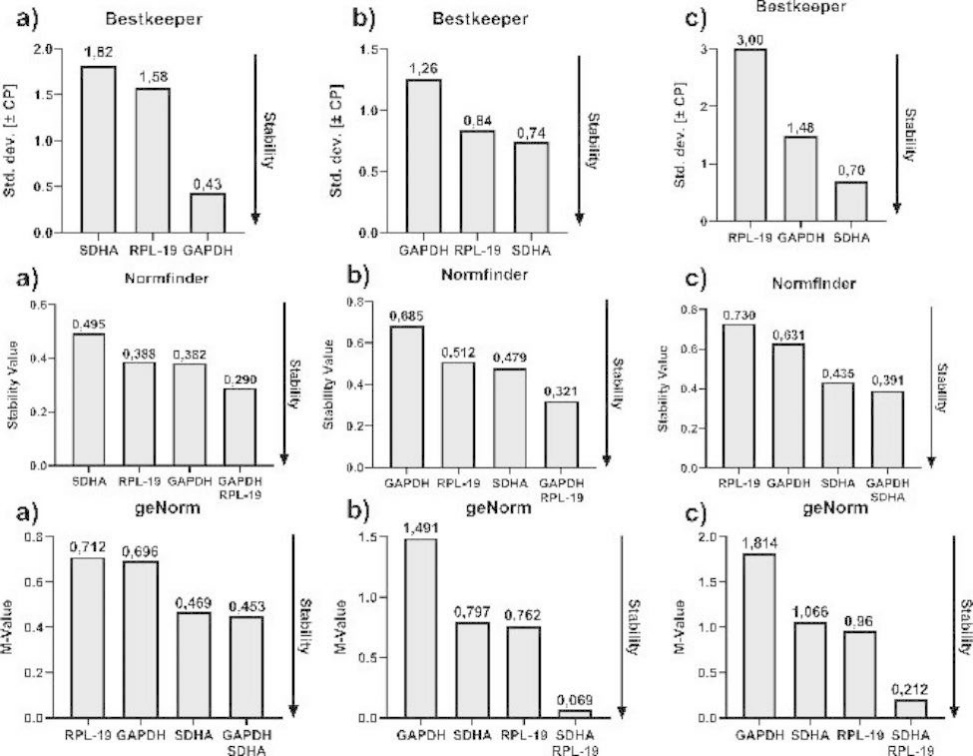
Stability test results calculated by three differents software programs (Bestkeeper, geNorm and Normfinder) for the Abomasum (**a**), Small intestine (**b**) and Large intestine (**c**)

With exception to the geNorm program, analyses using the other software programs (Bestkeeper and Normfinder) were similar to REST (Table 3) and expression profile values (Ct mean and CV) in different tissues shown previously (Fig. 2; Table 2). Bestkeeper and Normfinder indicated GAPDH in the abomasum and SDHA in the small and large intestines as the most stable genes in those tissues. The combination of GAPDH and SDHA in the abomasum and small intestine, and GAPDH and SDHA in the large intestine were also indicated by Normfinder. The geNorm analyzes indicated (except for GAPDH in the large intestine) that all other genes in all tissues were very stable (M < 1.5). However, the geNorm results were different from those obtained from the coefficient of variation between the analysed groups and other software programs. SDHA in the abomasum and RPL-19 in the small and large intestine were the most stable genes in those tissues. The combination between GAPDH and SDHA in the abomasum and SDHA and RPL-19 in the small and large intestine were also indicated (Fig. 3). In addition, a similiar and high coefficient of variation for SDHA (CV = 9.67) and RPL-19 (CV = 9.93) were observed in comparing them with the geNorm results in the abomasum, otherwise GAPDH showed the lowest coefficient of variation, but the software considered it as the second most stable. The coefficient of variation for SDHA (CV = 5.29) and RPL-19 (CV = 5.87) were similar in the small intestine, and the geNorm analysis indicated RPL-19 as the most stable and SDHA as the second most. Furthermore, RPL-19 showed the highest coefficient of variation (CV = 16.64) and SDHA had the lowest (CV = 4.12) in the large intestine, but the geNorm software showed RPL-19 as the most stable gene, SDHA as second and GAPDH (CV = 10.43) as the least stable (Table 2) (Fig. 3).

## Discussion

The best reference gene exhibits the most stable expression in different groups and tissues regardless of the experimental treatment applied [22, 23]. In this study, we evaluated GAPDH and RPL-19 sequence genes described by Zaros et al. in Nelore cattle, evolutionarily close to sheep, and the SDHA sequence gene described by. Smeed et al. in sheep of a temperate region infected by *Mycobacterium avium* with different paratuberculosis forms [16, 27]. Although many authors consider some reference genes as universal (such as GAPDH) [36–38], many studies have shown that they are never stable between different groups in different samples and experimental treatments [39–41]. For example, GAPDH is not indicated to be used in Bovine models due to lower stability in different experimental treatment tissues [12, 36–38], including in Zaros et al. for which the sequence gene was used in this study [16]. Two studies in Brazil evaluated reference gene expression in different ovine breeds resistant and susceptible to gastrointestinal nematodes and observed different results: Zaros et al. concluded that GAPDH expression was the least stable in all tissues of Somalis sheep, while Toscano et al. concluded that GAPDH expression was the most stable in Morada Nova sheep abomasum, which is similar to our results [17, 18].

In this study, RPL-19 was found to be the least stable reference gene in all tissues, which is different from the findings by Zaros et al. in cattle, in which these authors concluded it to be the best reference gene. RPL-19 is apparently a stable reference gene in bovines, independent from the infectious agent and degree of resistance to infection, as observed by Bricarello et al. in cattle resistant and susceptible to gastrointestinal nematode infections; and by Regitano et al. in cattle with and without tick infestation [12, 16, 30]. RPL-0 expression, another subunit member of the ribosomal protein family, was analyzed in two studies in Brazil with sheep, and was classified as stable in the abomasum but not in the small intestine, in which SDHA was the most stable [18].

The sequence described by Smeed et al. regarding SDHA in sheep with different paratuberculosis forms and used in this study was the most stable gene in the small and large intestine [27]. Furthermore, Zang et al. concluded that SDHA and GAPDH can be used as reference genes, but this was not observed in all tissues analyzed in our study [29].

SDHA was a reliable reference gene to use in sheep infected by *Mycobacterium avium* bacteria [42] and gastrointestinal nematodes, especially in the small intestine [24]. Other studies have also confirmed that SDHA was stable [28, 29]. Zaros et al. also evaluated the SDHA expression sequence described by Smeed et al. and obtained the same result as in our study [18, 28]. Again, a different infectious agent apparently does not influence gene expression, as observed in the studies by Bricarello et al. and Regitano et al. [16, 30]. Only two studies conducted in Brazil using different breeds and infection types evaluated reference gene expression in sheep resistant and susceptible to gastrointestinal nematodes, with both obtaining different results. Zaros et al. evaluated natural infection in Somalis sheep, while Toscano et al. evaluated experimental infection in Morada Nova sheep [17, 18].

This is the first study in Brazil to evaluate RPL-19 in tropical Northeast Brazilian sheep, showing stability in all tissues and the first to evaluate reference gene expression in the large intestine in sheep. We suggest that different breeds and climates can interfere in reference gene expression in ruminants, but not the kind of infectious agent. Just as in Zaros et al., we also demonstrated that there is no single universal reference gene for all tissues, but on the other hand this study and that by Zaros et al. were the only studies in Brazil which have analyzed reference genes previously reported in other studies with different conditions to be used in sheep from a semi-arid region in Northeast Brazil [18]. Thus, other studies developing similar methodologies using other genes with other variables such as ovine breeds, climates, infectious agents and evolutionary-close ruminants are necessary.

In conclusion, in these conditions evaluated, GAPDH was an ideal reference gene to be used to normalize gene expression studies in the abomasum, whereas SDHA was ideal in the small and large intestines in these sheep. These two genes were ranked as the most stable genes in our results using resistant and susceptible crossbreed sheep to being naturally infected by gastrointestinal nematodes to use in future immune response studies or other gene expression studies. Moreover, we demonstrated that without prior validation the reference genes already used in other studies are not possible to normalize the relative expression results due to different variables which can interfere in the results. We highlight the importance of using different software programs to analyze gene stability as they are essential to choose the most stable gene or the best association in order to avoid mistakes associated with relative expression analyzes. This was the first study in Northeast Brazil to evaluate the stability of RPL-19 in sheeps and the one of the few in Brazil to analyze reference genes in tropical sheep resistant and susceptible to gastrointestinal nematodes.

## Data Availability

All of the data generated or analyzed during this study are available from the corresponding author on reasonable request.
